# Improved Mass Calibration in MALDI MSI Using Neural Network-Based Recalibration

**DOI:** 10.1021/acs.analchem.4c00304

**Published:** 2024-05-06

**Authors:** Alexander Denker, Jens Behrmann, Tobias Boskamp

**Affiliations:** † Center for Industrial Mathematics, University of Bremen, Bibliothekstraße 5, Bremen, 28359, Germany; ‡ Bruker Daltonics GmbH, Fahrenheitstraße 4, Bremen, 28359, Germany

## Abstract

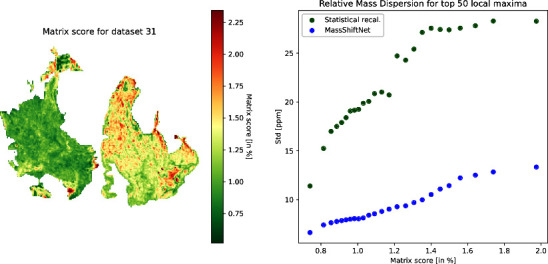

Matrix-assisted laser desorption/ionization mass spectrometry imaging (MALDI MSI) is a powerful imaging method for generating molecular maps of biological samples and has numerous applications in biomedical research. A key challenge in MALDI MSI is to reliably map observed mass peaks to theoretical masses, which can be difficult due to mass shifts that occur during the measurement process. In this paper, we propose MassShiftNet, a novel self-supervised learning framework for mass recalibration. We train a neural network on a data dependent and specifically augmented training data set to directly estimate and correct the mass shift in the observed spectra. In our evaluation, we show that this method is both able to reduce the absolute mass error and to increase the relative mass alignment between peptide MSI spectra acquired from FFPE-fixated tissue using a MALDI time-of-flight (TOF) instrument.

## Introduction

Matrix-assisted laser desorption/ionization mass spectrometry imaging (MALDI MSI) is a powerful technique that combines mass spectrometry and imaging capabilities to generate molecular maps of biological samples.^1^ By using a laser to desorb and ionize molecules in a sample, MALDI MSI allows for the detection and spatial localization of biomolecules, such as peptides or lipids, in tissues or cells. The technology has numerous applications in various fields in the life sciences, such as cancer research, neurobiology, and drug development. Many of these applications depend on reliable mapping of observed mass peaks to theoretical masses, which can be challenging due to mass shifts occurring in the measurement process. Thus, mass calibration is often the first step in any analysis of the MALDI MSI data.

MALDI MSI calibration methods can be generally classified into external and internal methods. In external methods, a known calibrant is deposited outside of the tissue area. By comparing the observed spectra to the known calibrant composition, one can estimate a mass shift function, which is then applied to calibrate all spectra in the MSI data set. However, external calibration cannot account for local mass shifts occurring inside the tissue area caused by, for example, inappropriate height correction or inhomogeneous space charge effects. The latter is a common issue, in particular, when using MALDI time-of-flight (TOF) instruments.

For internal methods a known calibrant is sprayed onto the tissue and measured together with the tissue, thus allowing to capture local mass shift variations. However, the internal calibrant may impact the ionization of tissue molecules and thus may reduce the data quality. In some recent works, a-priori knowledge about features or molecules in measurements has been used as internal calibrants. For example, the presence of typical molecules^2^ or theoretically known matrix peaks^3^ can be used to estimate the mass shift and calibrate the spectrum.

Boskamp et al.^4,5^ leverage a-priori information about the analytes to be measured and propose a recalibration method based on statistical properties of the chemical noise background in peptide imaging experiments. This method, which we refer to as *statistical recalibration* throughout this work, maps the spectra to the Kendrick mass scale and estimates the mass shift by comparing statistical properties of the Kendrick mass defect with expected theoretical features. However, this method is limited to measurements of peptides or similar analyte classes and cannot deal with more complex mixtures, such as, for example, lipids. Moreover, the accuracy of this method degrades in the presence of strong matrix signals, which often cannot be avoided. As an improvement over this method, we propose a neural network approach, referred to as MassShiftNet, to learn relevant features from Kendrick scale representations of a suitable training data set.

Deep learning methods have shown remarkable success in extracting features from large scale data sets and computing predictions on these learned features.^6^ However, the successful application of supervised deep learning relies on the availability of large labeled data sets. The application of supervised deep learning to mass calibration has been limited by this constraint, as there are no appropriate labeled data sets available, i.e., uncalibrated spectra and corresponding mass shift functions. We tackle this problem by constructing a data set of calibrated spectra directly from the data, augmented by simulated mass shifts. This construction relies on an auxiliary model-based recalibration method that approximates a measured spectrum by a composition of different signal components according to theoretical models of analyte and matrix molecules. Even though this auxiliary method is computationally demanding, the combination with the aforementioned deep learning approach yields a recalibration algorithm that is suitable for large scale MALDI MSI data sets.

The paper is structured as follows. We start by introducing the statistical recalibration method, i.e., the idea of leveraging Kendrick mass defect maps proposed earlier for mass recalibration,^4^ which will serve as the baseline method to compare to. We then present the construction of the training data set and the architecture of the MassShiftNet, and finally introduce the auxiliary, model-based recalibration algorithm and how it can be used for constructing the training data set.

In the last part of the paper, we evaluate both the model-based approximation and the MassShiftNet on a collection of 31 FFPE tissue samples of peptide MSI measurements acquired on a MALDI TOF instrument. By combining the supervised deep learning method with an automated, data-driven generation of training data, we empirically show that the neural network is able to improve the relative mass alignment between spectra as well as to reduce the absolute mass error.

## Materials and Methods

In this section, we will present our self-supervised learning approach MassShiftNet to estimate and correct mass shifts in MALDI MSI measurements. In order to obtain a suitable training data set, we introduce an auxiliary, model-based recalibration algorithm, based on comparing the observed spectra to theoretical reference spectra generated using prior knowledge of the analytes and matrix under consideration.

### MALDI MSI Data Acquisition and Processing

We evaluate our method on the same cohort of *N* = 31 FFPE tissue samples used in Boskamp et al.,^4^ obtained at three different laboratories: the Center for Histology, Cytology, and Molecular Diagnostic in Trier, the University Hospital Heidelberg, and the University Hospital Carl Gustav Carus in Dresden. The collection of samples adhered to the ethical guidelines of the respective local ethics committees. For details about the sample preparation we refer to.^4^ MALDI MSI data sets were acquired using a rapifleX Tissuetyper TOF instrument (Bruker) in positive-ion reflector mode. Mass spectra were collected in the *m*/*z* range 600–3200 at 100 μm spacing between spot centers. External calibration was done using Peptide Calibration Standard II (Bruker). Mass resolving power was approximately *R* = 40000 (manufacturer specs at 3.2 kDa). The data was loaded using the SCiLS Lab software (version 2023b, Bruker Daltonics, Bremen, Germany) and processed using the SCiLS Lab API and self-written Python scripts. For comparison to the recalibration method of Boskamp et al.^4^ (in this paper referred to as *statistical recalibration*), we used the implementation of Deininger et al.^5^

### Kendrick Mass Defect Map

The Kendrick mass defect map of a spectrum describes the mass defects of observed molecular masses relative to a suitably chosen Kendrick scale.^4,7^ The Kendrick scale is chosen appropriately according to the considered molecular class.

For a given molecular mass *m* we define the (centered) Kendrick mass defect to base λ as
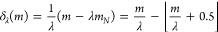
1where ⌊*x*⌋ denotes the floor function and *m*_*N*_ is the nearest nominal mass given by . Note that by this definition, the centered Kendrick mass defect is always in the range of [−0.5, 0.5]. Evaluating the Kendrick mass defect for all observed masses *m* in a spectrum defines the mass defect map (*m*, δ_λ_(*m*)).

**Figure 1:**
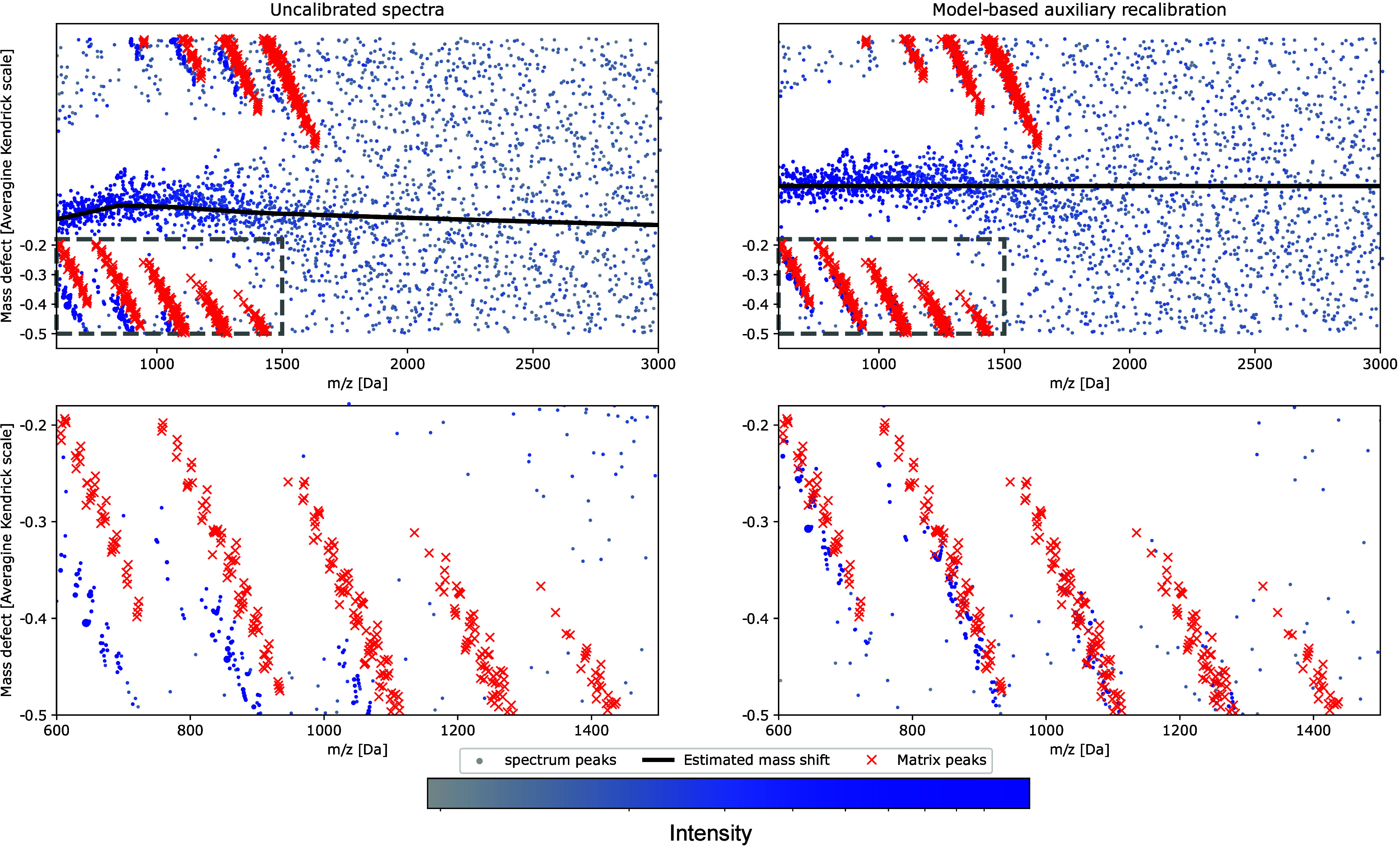
Kendrick mass defect map for a single spectrum before (left) and after model-based recalibration (right). Signal peaks (blue and gray dots, colors indicate peak intensities) form large scale structures corresponding to peptide and matrix components. The mass shift function (black line) is estimated from the location of the peptide component. The comparison to the theoretical matrix peak locations (red crosses) confirms the improved mass accuracy after calibration (close-up plots, bottom). Note that due to the strong matrix content in this spectrum the peptide signal is only observed up to approximately 1500 *m*/*z* and disappears in the noise in the higher mass range.

The mass defect map has a specific structure that depends on the type of analyte under consideration. Deviations of this structure can be attributed to mass shifts, and thus, the mass shift function can be estimated using the mass defect map. In Figure 1 the mass defect map is shown for peptide measurements, where the deviations from 0 can directly be interpreted as the mass shift. For these analytes, the averagine peptide model^8^ can be employed, which is based on the observation that peptides are clustered around equidistant masses with a spacing of a little more than 1 Da. The exact mass *m* of a peptide is approximately proportional to its nominal mass *m*_*N*_, i.e., *m* ≈ λ_av_*m*_*N*_, with λ_av_ ≈ 1 + 4.95 × 10^–4^, and, as a consequence, δ_λ_(*m*) ≈ 0. Thus, for observed spectra, the location of the cluster formed by peptide masses in the Kendrick defect map directly corresponds to the effective mass shift function.

In case a spectrum is dominated by peptide signals, extracting the mass shift function from the Kendrick mass defect is straightforward.^4^ In the presence of a strong matrix signal, however, or in the case of other classes of analytes showing more complex patterns in the Kendrick mass defect map, the effective mass shift is harder to analyze from the mass defect map.

Note that the Kendrick mass defect map as defined above differs from the conventional Kendrick diagram in that it uses the centered Kendrick mass defect, whereas the classical Kendrick mass defect is a quantity in [0, 1]. Moreover, the conventional Kendrick diagram is typically used on data with high mass resolution and accuracy, allowing us to infer chemical properties of observed ion species from the location of their spectral signals in the diagram. In the present context, the Kendrick mass defect map serves a different purpose, as we are interested in statistical properties of the mass defects observed in a spectrum and in the larger structures becoming apparent in its visualization.

As in the statistical recalibration approach, we represent the mass defect map as a 2D histogram *H* over the mass and mass defect axes, accumulating the spectral intensities at mass *m* in the 2D histogram bin containing the point (*m*, δ_λ_(*m*)). (Strictly speaking, a mass spectrum consists of spectral intensities for observed mass over charge ratios *m*/*z* and does not directly reveal the mass of a detected molecule. In MALDI MSI, however, single-charged ions (*z* = 1) are detected almost exclusively, hence we can identify mass and *m*/*z*.) This representation is more robust in the presence of strong noise, as typically observed in single spectra, and at the same time allows us to encode the mass defect map by a fixed number of variables, allowing it to be used as the input to a neural network, as described in the next section.

### Learning the Mass Shift from the Mass Defect Map

Our goal is to estimate the mass shift function of a spectrum from the underlying structure apparent in the spectrum’s mass defect map. Instead of fitting a statistical model of the analyte and matrix mixture under consideration to the mass defect map, we employ self-supervised learning techniques to learn these patterns from a suitably generated and augmented training data set.

The complete learning process consists of three steps: (1) obtain a small set of calibrated spectra, (2) apply simulated mass shift functions to these spectra, and (3) use the mass defect maps of the distorted spectra (represented by histograms *H*) and the corresponding mass shift functions  as training data for the MassShiftNet neural network.

In the first step, we take a random selection of spectra from the same or a similar MSI data set and apply an auxiliary recalibration technique to obtain a set of calibrated spectra. In principle, any recalibration technique can be employed here. In our work, we use a model-based decomposition algorithm, which is presented in the next section. Since this algorithm is computationally demanding, it is applied only to a relatively small number of spectra (150 in our experiments).

For the second step, we simulate random mass shift functions  and apply these to the calibrated spectra to obtain distorted, uncalibrated spectra with known mass shifts. The simulated mass shift functions are chosen to resemble mass shifts often seen in real MSI measurements. In our experiments, we use linear functions, Δ(*t*) = β_1_*t* + β_2_, and square root functions, , with random parameters β_1_ and β_2_, since these functions resemble the type of mass distortions observed in data from MALDI TOF instruments. Further, we employed scaled and shifted versions of the mass shift functions that were estimated from the observed spectra in the first step. This allows us to simulate a large number of distorted spectra from one calibrated spectrum and create a training data set {*H*_*i*_, Δ_*i*_} for *i* = 1, ..., *N* of histograms *H*_*i*_ and simulated mass shift functions Δ_*i*_. In the last step, we train the MassShiftNet *f*_θ_ by minimizing the mean squared error of the network output,
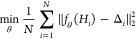
2

Once the network is trained, only one forward pass is needed to estimate the mass shift function for each spectrum in the MSI data set. In this way, the trained network can be used as a fast recalibration technique.

### MassShiftNet Network Architecture

An important aspect of our approach is selecting a suitable neural network architecture for the MassShiftNet. The input to the network is a histogram *H* of size *L* × *K*, where *K* is the number of *m*/*z* bins and *L* the number of Kendrick mass defect bins. Since this histogram can be interpreted as an image, we can make use of convolutional neural networks, which are very successful for a wide variety of imaging problems.^6,9,10^ In particular, we propose a network architecture that consists of an initial feature extraction block with several convolutional layers, followed by an additional fully connected prediction network. We provide a schematic overview of the network architecture in Figure S3 in the Supporting Information.

#### Feature Extraction

The feature extraction part is composed of two components, each serving a specific purpose in processing information from the input histogram. The first component employs a series of 1D convolutional layers that operate individually on each *m*/*z* bin in the histogram. For this, the histogram is split into columns, representing the *m*/*z* bins. For each column, a different set of convolution filters is learned. Each 1D convolution integrates circular padding to take into account the circular structure of the Kendrick mass defect: Mass defects larger than 0.5 wrap around the other side to a defect of −0.5 at a higher mass value. After each 1D convolutional layer we use 1D batch normalization^11^ and a LeakyReLU activation function. In this first part of the network, each *m*/*z* bin is processed on its own. This mimics the processing of the histograms in the statistical recalibration approach.^4^ All in all, this component consists of six 1D convolutional layers with batch normalization and activation functions. In the second component we use 2D convolutions, which allows information to be exchanged between *m*/*z* bins. Here, we use 2D batch normalization and LeakyReLU activation functions after each 2D convolutional layer. This block consists of four 2D convolutional layers with batch normalization and activation functions.

#### Prediction Network

In this block, we first apply an initial 1D convolution to reduce the number of channels back to 1. Similar to the first step in the feature extraction part, the processed histogram is split into *Km*/*z* bins. For each *m*/*z* bin we use a multilayer perceptron (MLP) to produce the final mass shift estimation for the bin. Each MLP is implemented as a fully connected layer with LeakyReLU activation functions. For the final output, we use a tanh activation function scaled by 0.5 to ensure the output is always in the interval [−0.5, 0.5].

### Model-Based Auxiliary Recalibration

For training the MassShiftNet neural network, we needed to create a suitable set of training data. As discussed, this requires the use of an auxiliary recalibration method for obtaining a small set of calibrated spectra. The model-based recalibration method proposed here is based on a decomposition of the spectra into multiple components corresponding to the expected mixture of the different analytes under consideration. Assuming an appropriate theoretical model of the analyte and matrix signal components, we then aligned the observed spectra to best fit the theoretical model. By minimizing a particular loss function, we simultaneously estimate the mass shift and baseline functions as well as spectral intensities of the various model components. A schematic flowchart of this method is given in Figure S4 in the Supporting Information.

In the context of our validation study, the observed spectra mainly consist of peptide and CHCA matrix signals. We represent the peptide components using the averagine model,^8^ and we use a combinatorial model for the exact masses of the CHCA matrix clusters,^12^ incorporating the different expected adducts and isotopes for a range of cluster sizes.

More specifically, we consider a spectrum *X*(*t*) on the *m*/*z*-interval [*m*_min_, *m*_max_] consisting of intensities *X*(*t*_*k*_) at discrete *m*/*z* locations (*t*_*k*_, *k* = 1, ..., *d*). The theoretical model consists of components (*S*_*i*_, *i* = 1, ..., *N*), each corresponding to a list of potential peak locations (*p*_*j*_^*i*^ ∈ [*m*_min_, *m*_max_]: *j* = 1, ..., *M*). If the actual mass shift in the observed spectrum *X* is small, we can approximate it by a mixture of model components,
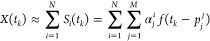
3where each spectral peak is modeled by a suitable elementary peak function *f*(*t*), typically a Gaussian with an appropriately selected peak width (σ = 0.05 Da in our experiments). The α_*j*_^*i*^ parameters represent the individual peak intensities.

In order to capture the mass shifts observed in the spectrum *X*, we extend the model by introducing a mass shift function Δ_ψ_(*t*) parametrized by a parameter vector ψ. Similarly, we add a baseline function *b*_ϕ_(*t*) to the model that allows the approximated baseline effects often visible in MALDI TOF measurements.

This leads to the optimization problem
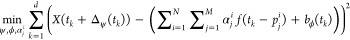
4aiming to find intensity parameters α_*j*_^*i*^, as well as mass shift and baseline parameter vectors ψ and ϕ describing an optimal approximation of the model to the observed spectrum.

Note that instead of matching the observed peaks with expected reference peaks, as in common recalibration methods, we model the complete spectrum, including the individual peak intensity values α_*j*_^*i*^. This allows us to consider a large number of potentially expected reference peaks, many of which may not be detectable in the observed spectrum *X*, and thus are assigned a zero intensity, α_*j*_^*i*^ = 0. On the other hand, methods that only rely on matching *m*/*z* values for recalibration can be highly sensitive to outliers or spurious peak matches.^13^

Moreover, existing methods often represent the mass shift function Δ_ψ_ as linear^14^ or piecewise linear functions.^13^ In contrast, we parametrize both the mass shift function Δ_ψ_(*t*) and the baseline function *b*_ϕ_(*t*) as feed-forward neural networks.^6^ Neural networks have been proven to be particularly powerful and flexible for function approximation. They have been used for representing images or audio data,^15^ 3D scenes,^16^ and even for solving differential equations.^17^ Here, we are using a feed-forward neural network with one hidden layer, ReLU activation functions and the final activation omitted, which allows approximating more complex, nonlinear mass shift and baseline functions. However, as this requires solving the optimization problem for each spectrum to be calibrated, the method is much slower, compared to other methods that rely on simpler summary statistics. An acceleration can be achieved by initializing the neural network parameters from the previously processed spectrum, but applying this method to all spectra in a typical MALDI MSI measurement is infeasible.

Instead, as mentioned above, we apply this method only to a small subset of spectra, thus obtaining calibrated spectra that are used for generating the training data for MassShiftNet. Moreover, the model approximation yields a decomposition of the spectrum into different components, in our case, peptide and matrix signals. This allows us to perform additional training data augmentation by reweighting the different components, e.g., increasing or decreasing the relative intensity of the matrix signal, enabling us to generate a broad and realistic data set for the supervised training of the MassShiftNet.

## Results and Discussion

We tested our method on the set of 31 FFPE tissue samples of peptide measurements. Due to the absence of ground truth data containing perfectly aligned spectra, evaluating the precision of mass shift calibration methods poses a challenge. To overcome these limitations, we employ two distinct quality metrics to assess the effectiveness of our recalibration technique. We report the relative mass dispersion, i.e., the standard deviation of peak locations, for the 50 highest peaks of the mean spectrum for each MSI data set. In addition, we report the absolute mass shift of selected known matrix peaks. For the latter, we use the aforementioned combinatorial model for matrix molecules,^12^ thus being able to measure the distance of a measured peak to the closest theoretical matrix peak. This allows us to estimate the absolute mass accuracy without having to require any particular peptide analyte to be abundant throughout all of the samples. As we compare our methods to the statistical recalibration method proposed earlier,^4^ we use the same calculations to estimate the relative mass dispersion. The absolute mass shift is computed with respect to nine manually selected matrix peaks that appear at high intensity in all MSI data sets.

### Training of the MassShiftNet

We train an individual MassShiftNet for each of the 31 MSI data sets. For each MSI data set, we use the model-based auxiliary recalibration method to calibrate spectra at 150 randomly chosen spots. For each calibrated spot, we created 2500 randomly generated mass shift functions. For 60% of these mass shift functions, we use random linear shifts, for 10% random square root shifts, and for the remaining 30% we generate perturbations of the 150 extracted shift functions. In addition, at every training iteration, we vary the overall matrix signal intensity by adding between 0 and 100% of the matrix spectrum extracted by the model-based auxiliary recalibration.

We create mass shift histograms *H* using 24 bins for the *m*/*z* axis and 256 bins for the Kendrick shift axis δ. Both the model-based auxiliary approach and the MassShiftNet were implemented using Pytorch^18^ to support GPU processing. We train using the Adam optimizer^19^ with a learning rate of 10^–4^ and a batch size of 64.

### Relative Mass Dispersion

We follow the experimental setting proposed in Boskamp et al.^4^ For each MSI data set, the following steps are performed: First, the 50 most intense local maxima of the mean spectrum are collected using a window of 3 Da. For each of these top 50 peaks, we compute the nearest peaks in all individual spectra by matching pursuit using a Gaussian (fwhm = 0.15 Da, equiv to σ = 0.0637 Da). These peak candidates are further filtered using a threshold of 2.5 times the respective background intensity, where the background intensity is obtained by dividing a 200 Da range around the candidate into 1.1 Da windows and taking the median of the maximum intensity in each window. In an additional filtering step, only peaks with a coverage (i.e., the percentage of spectra in which this peak was found) of at least 10% were considered for the final evaluation. We then compute the mass dispersion of each peak as the standard deviation of the peak locations across all of the spectra.

**Figure 2:**
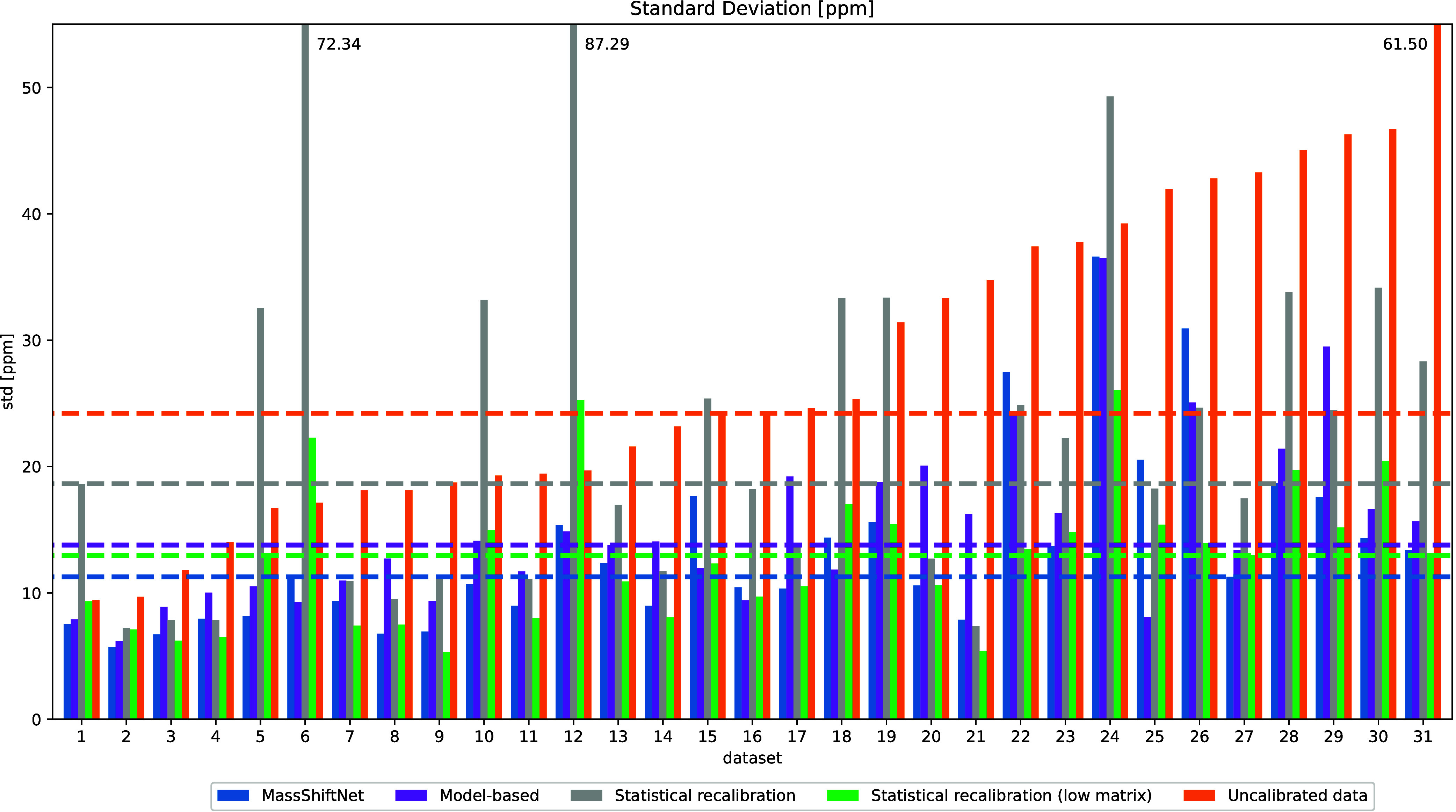
Relative mass dispersion of the top 50 local maxima of the raw mean spectrum. The dashed lines represent the median standard deviation over all 31 MSI data sets.

We present the median mass dispersion of the top 50 peaks per data set in Figure 2, comparing the MassShiftNet to the model-based auxiliary approach, the statistical recalibration approach, and the uncalibrated signal. As the statistical recalibration method was only developed for spectra with a sufficiently low matrix signal,^4^ we additionally evaluate the statistical recalibration on the subset of spots with low matrix signal. In order to define this subset, we extracted four *m*/*z* values (*m*/*z* 644.08, 650.10, 682.09, and 855.10 Da) corresponding to matrix peaks and computed the sum of the intensities of these four peaks for each spot. A spectrum is considered having a low matrix signal if this summed intensity is less than a threshold defined as 60% of the 99% quantile across all spectra. The model-based auxiliary recalibration method, on the other hand, is evaluated on only 10% of the spots (evenly distributed across the slide), due to the high computational cost of this algorithm.

The misalignment seen in the uncalibrated data (ranging from 8.0 to 61.5 ppm, median 24.1 ppm) is representative for an instrument of the given mass resolution (*R* = 40000) and protocols using external calibration. Using internal calibration, a misalignment of approximately 10 ppm is to be expected under ideal conditions. The MassShiftNet comes close to this ideal limit and achieves the lowest mass dispersion of 11.27 ppm. The statistical recalibration evaluated on low matrix spectra yields a mass dispersion of 12.96 ppm and thus performs slightly better than the model-based approach (13.78 ppm). These differences, however, are relatively small and not necessarily significant.

When the statistical recalibration is evaluated on all spectra, on the other hand, the mass dispersion increases to 18.64 ppm. Thus, in contrast to the statistical recalibration, the model-based approach is able to deal with spectra containing strong matrix signals, as shown in Figure 1 for one example spectrum. This increased robustness allows to apply the method to blindly selected spectra for generating the training data, thus also increasing the robustness of the MassShiftNet method.

### Absolute Mass Error

**Figure 3:**
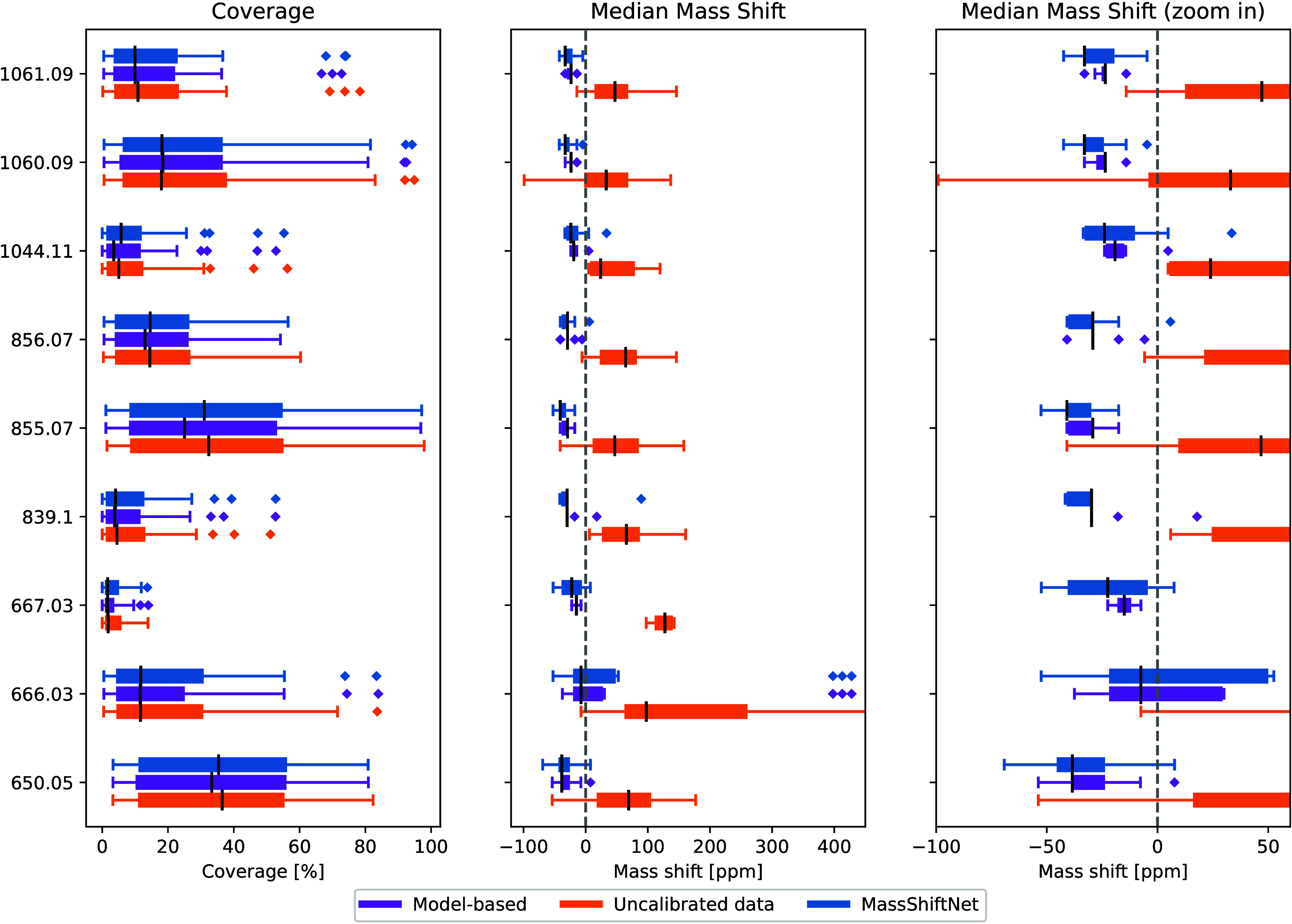
Coverage and median mass shift of known matrix peaks for the uncalibrated data, as well as for data recalibrated using the model-based and the MassShiftNet methods. Both methods are consistently able to reduce the overall mass shift.

In order to evaluate the reduction in the absolute mass error, we evaluate the mass shift between measured matrix peaks and their known exact masses for different recalibration methods. We manually picked nine matrix peaks between *m*/*z* 650 and 1100 with a relatively high intensity in all MSI data sets. In order to find the peaks nearest to the expected exact masses, we used the same peak picking approach as for the relative mass dispersion. We limited the evaluation to matrix peaks with at least 10% coverage in 10% of the data sets, which excluded the matrix peak at *m*/*z* 667.03 that had a coverage below this threshold. For the remaining eight matrix peaks, the median mass shifts over all spectra in each data set are shown in Figure 3. In this comparison, the model-based recalibration yields the lowest mean median mass shift (i.e., the mean over the eight matrix peaks) of −23.94 ppm vs −28.70 ppm for the MassShiftNet and 63.88 ppm for the uncalibrated data. Additional results for the root mean squared error are given in the Supporting Information. Again, the obtained mass accuracies are comparable to what has been reported for other methods^4,14^ and come close to what may be expected under ideal conditions. The differences between the two calibration methods are comparably small. The minor advantage on the side of the model-based method may be due to the fact that this method makes explicit use of a chemical model describing the matrix cluster molecules, whereas for the MassShiftNet method, this a-priori knowledge is only implicitly encoded in the trained neural network.

The numbers above refer to the mass accuracy of the matrix signals in all spectra acquired from the measurement region. Since the physical effects giving rise to mass inaccuracies affect different molecule classes independently of their chemical composition, the absolute mass errors of peptide analytes can be assumed to be of approximately the same size as those observed in matrix molecules, at least for peptides in the lower mass range up to 1100 Da.

### Matrix Score

The model-based recalibration, as described above, includes a decomposition of the spectra into a matrix and an analyte component (peptides in our examples). This decomposition allows us to define the matrix score for each spectrum, i.e., the relative contribution of matrix clusters to the overall signal. Since a high matrix score indicates a relatively low analyte signal, this score may serve as a quality metric, indicating regions where little or no analyte ions were detected.

More specifically, model approximation (eq 3) represents a decomposition of a spectrum *X*(*t*) into the analyte signal *S*_1_(*t*) and the matrix signal *S*_2_(*t*). This decomposition can be used to define the matrix score as the total intensity of the matrix signal relative to the overall spectrum intensity,
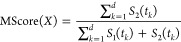
5

**Figure 4:**
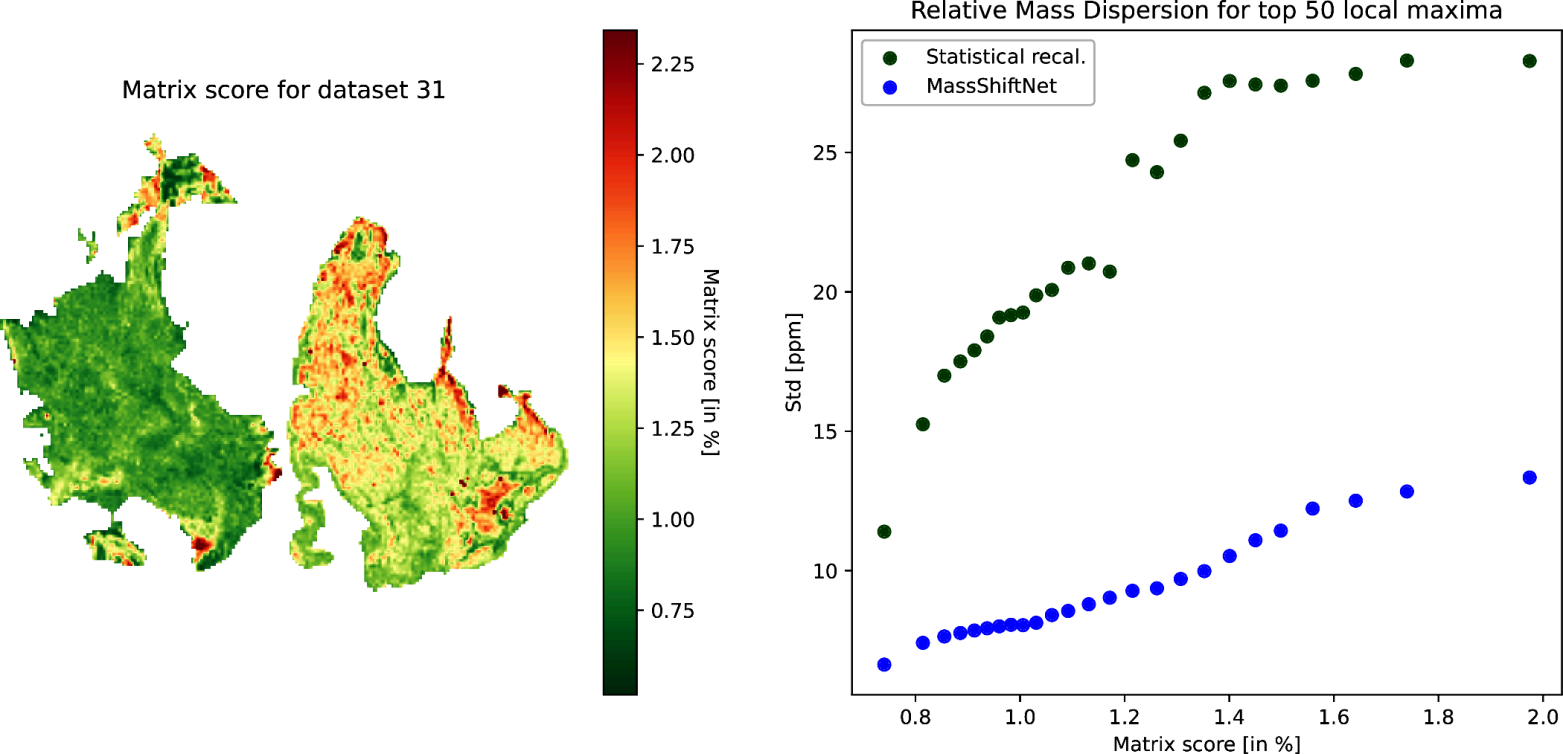
Left: Distribution of the matrix score for data set id 31. The matrix score is given in percent of the total intensity per spot. Right: Relative mass dispersion depending on the matrix score threshold.

The matrix score can be computed for each spot in the MSI data set and may help to assess the uniformity of the matrix on the sample. In Figure 4 we show the matrix score image for data set 31 (cf. Figure 2).

The matrix score can be used to investigate how the matrix signal intensity impacts the performance of the different recalibration methods. For this purpose, we compute the median relative mass dispersion as above but are limited to spectra with a matrix score less than a specific threshold (Figure 4, right). It can be seen that the performance of the statistical recalibration method strongly decreases as the threshold is increased. The performance of the MassShiftNet method, on the other hand, exhibits a much smaller dependency on the matrix score threshold.

### Computation Time

For practical applications of a recalibration method, computation time is a crucial factor. In our evaluation, the statistical recalibration method required 0.08 s per spot on average when using the implementation of Deininger et al.^5^ (Intel Core i7–4790 3.60Ghz). The model-based recalibration, on the other hand, took 3.3 s per spot even using GPU hardware acceleration (GeForce RTX 2080), which is approximately 40 times longer. In contrast, the mass calibration with the trained MassShiftNet only took 0.039 s per spot on the same GPU. This does not include the training of the MassShiftNet, which took approximately 40 min per data set (3 min training data generation and 37 min training). The overall average calibration time per spot depends on the size of the data set. For example, with data set 0 consisting of 33845 spots, the total processing time per spot is 0.11 s, which is comparable to the statistical recalibration technique.

## Conclusion

In this work, we presented the MassShiftNet method, a novel mass recalibration approach based on a deep learning neural network trained in a self-supervised manner. The design of MassShiftNet utilizes the concept of Kendrick mass defect analysis, thus exploiting the intrinsic structures hidden in the spectral data that are made apparent by this concept.

The training data set for the MassShiftNet network is constructed by using an auxiliary, model-based recalibration method that reliably estimates a spectrum’s mass shift function. Applying this method to a small set of real, observed spectra and combining the calibrated spectra with a wide variety of simulated mass shift functions yields a training data set suitable for training the neural network and appropriate for the MSI data set under investigation. An alternative approach of generating a fully synthetic training set, including simulating the underlying spectra, may help to avoid the time-consuming model-based spectra recalibration but is beyond the scope of this work.

In our evaluation, we applied the proposed method to peptide MSI, which allowed us to compare it to the previously presented statistical recalibration method, which is explicitly limited to this class of molecules. However, our new approach can easily be extended to other classes of molecules that exhibit characteristic structures in a Kendrick mass defect map, as long as a chemical model or a-priori knowledge about expected peak locations is available. Extensions to such further molecular classes will be the subject of future work. These will then also allow one to more directly assess the absolute mass error in the acquired MSI data by utilizing the spectral features of well described endogenous analytes (e.g., lipids).

In our experiments, we trained the MassShiftNet for each MSI data set separately. Depending on the context, it may alternatively be desirable to train the neural network only once and then apply it to a series of data sets. We have performed a preliminary evaluation of this scenario by applying the trained MassShiftNet from one data set to all other data sets, thus testing the network’s generalization capabilities. As expected, the median relative mass dispersion increases only moderately by approximately 4 ppm (see further details in the Supporting Information S2). Further experiments are needed to systematically evaluate the MassShiftNet’s generalization capabilities.

We have evaluated the proposed method on data from an axial TOF instrument as this class of instruments is known to be most affected by inaccurate mass measurements. In principle, our method is also applicable to data from other types of instruments, where mass misalignment may also be a concern, albeit to a lesser degree. It still needs to be investigated, however, whether the proposed method would actually allow further improvement of this type of data, beyond the level of mass accuracy already provided by the instrument itself.

## Supporting Information Available

Figure S1: Additional results of the root-mean-squared error of matrix peaks. Figure S2: Evaluation of the generalization capabilities. Results of the relative mass dispersion over top 50 local maxima. Figure S3: Overview of the network architecture. Figure S4: Flowchart of the model-based recalibration method (PDF)
