# Supplementary material for: Improved Mass Calibration in MALDI MSI Using Neural Network-Based Recalibration
Source: Anal Chem. 2024 May 6;96(19):7542–9. doi: 10.1021/acs.analchem.4c00304 (PMC11099886; doi:10.1021/acs.analchem.4c00304)
Supplement: Supplementary file 1 — ac4c00304_si_001.pdf [file ac4c00304_si_001.pdf]

Improved Mass Calibration in MALDI MSI using Neural Network-based Recalibration
Supporting Information

Alexander Denker(1), Jens Behrmann(1), Tobias Boskamp(2)
(1)Center for Industrial Mathematics, University of Bremen,
28359 Bremen, Bibliothekstraße 5, Germany
(2)Bruker Daltonics GmbH, 28359 Bremen, Fahrenheitstraße 4, Germany

Abstract

This is an appendix to the manuscript *Improved Mass Calibration in MALDI MSI using Neural Network-based Recalibration* presenting additional results of our proposed recalibration approach. We show the absolute mass error, evaluate generalization capabilities and show a visualization of the MassShiftNet architecture. Further, we provide a flow chart of the model-based recalibration method.

Contents

S1 Additional Results: Absolute Mass Error 2

S2 Generalization of MassShiftNet 3

S3 MassShiftNet architecture 4

S4 Model-based recalibration 5

S1 Additional Results: Absolute Mass Error

We additionally present the root mean square (RMS) of the mass error for the different methods, including the statistical recalibration, the auxiliary, model-based recalibration, and the MassShiftNet method. Note that the coverage threshold of the manually selected matrix peaks was only satisfied in 25 out of the 31 MSI datasets.

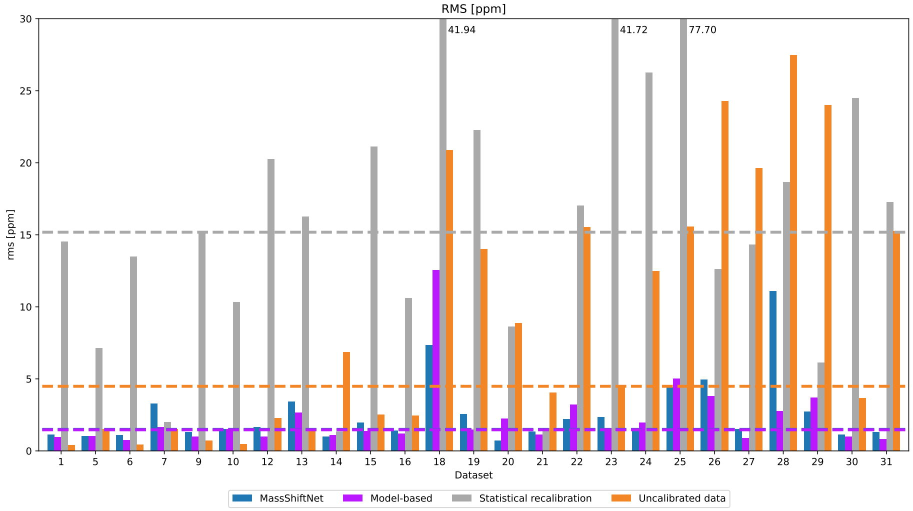

Figure S1: Root mean squared error (RMS) of eight manually selected matrix peaks. The dashed lines represent the median RMS over all MSI datasets.

S2 Additional Results: Generalization of MassShift-Net

In order to test the generalization capabilities of the MassShiftNet, we applied MassShiftNet trained on dataset 18 (chosen randomly) to all other datasets and evaluated the relative mass dispersion. The results are presented in Figure S2. The median relative mass dispersion drops from 11.27 ppm to 15.65 ppm. However, there are also some datasets (datasets $22, 24, 25, 26$) on which the pre-trained MassShiftNet achieved a better relative mass dispersion.

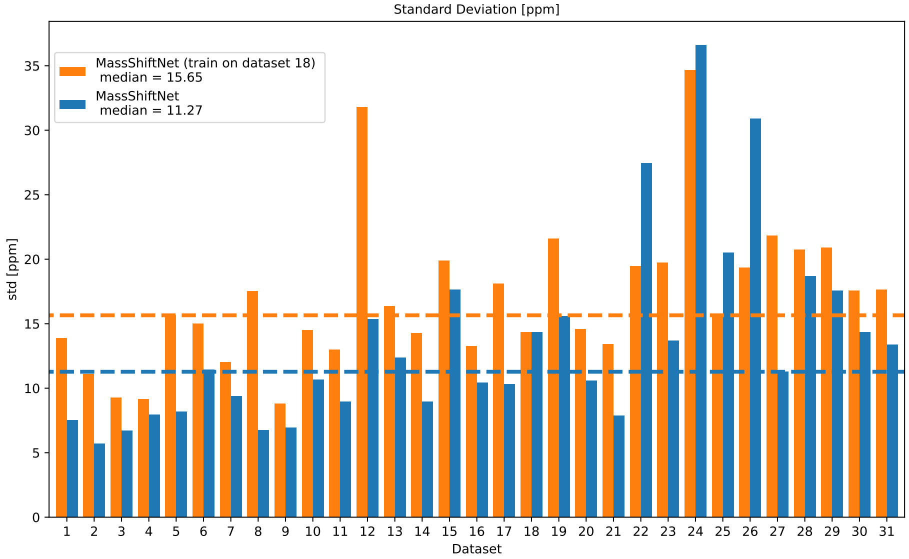

Figure S2: Relative mass dispersion of the top 50 local maxima. We compare the training setting in the paper (blue) with a MassShiftNet trained only on dataset 18 (orange).

S3 MassShiftNet architecture

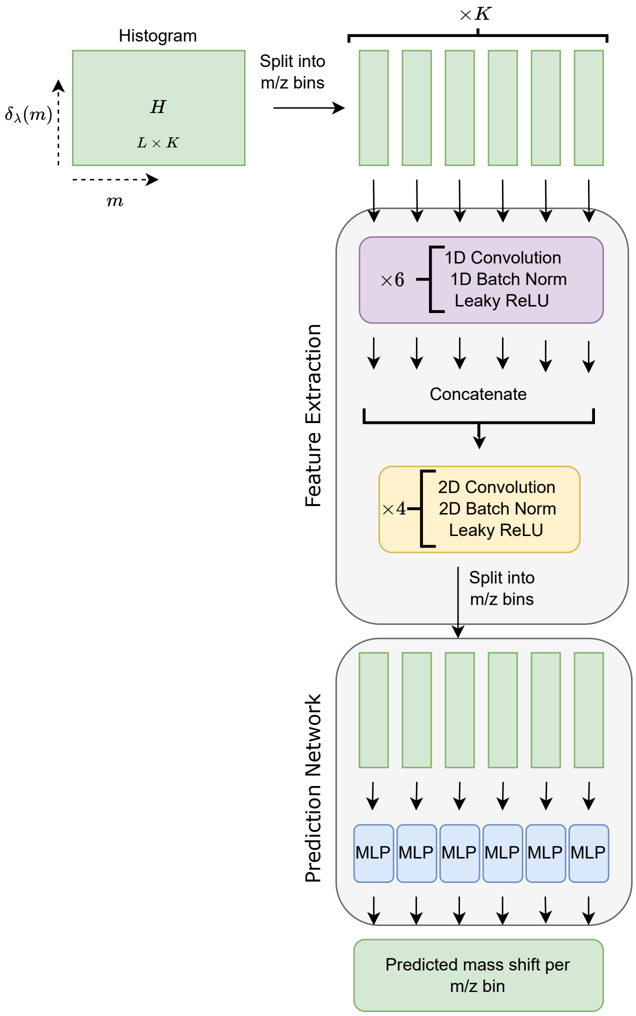

Figure S3: Schematic overview of the proposed MassShiftNet.

S4 Model-based recalibration

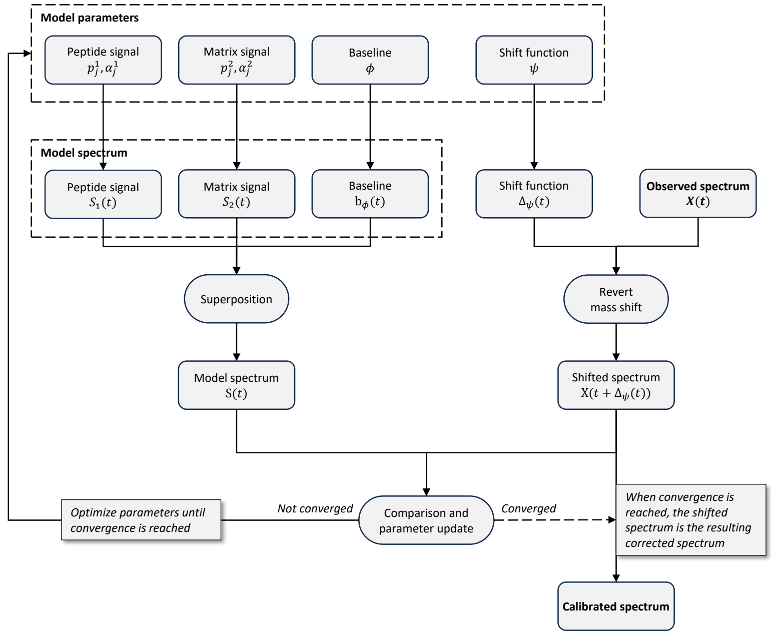

Figure S4: Flow chart of the model-based recalibration scheme. From a given set of model parameters, the model spectrum, consisting of peptide, matrix, and baseline components, as well as a mass shift function is formed. Using the mass shift function, the mass shifts in the observed spectrum are reverted. The result is compared to the model spectrum, and all model parameters are updated to reduce the difference. When convergence is reached, the input spectrum is calibrated.
